# Diagnostic performance of imaging investigations in detecting and differentiating cardiac amyloidosis: a systematic review and meta‐analysis

**DOI:** 10.1002/ehf2.12511

**Published:** 2019-09-05

**Authors:** Jack Brownrigg, Massimiliano Lorenzini, Matthew Lumley, Perry Elliott

**Affiliations:** ^1^ Pfizer Limited Walton Oaks, Dorking Road, Walton‐on‐the‐Hill, Tadworth Surrey KT20 7NS UK; ^2^ Barts Heart Centre St Bartholomew's Hospital London UK

**Keywords:** Cardiac amyloidosis, Transthyretin amyloid cardiomyopathy, Light‐chain amyloidosis, Heart failure, Nuclear scintigraphy, Cardiac magnetic resonance

## Abstract

**Aims:**

The study aims to systematically assess the diagnostic performance of cardiac magnetic resonance (CMR) and nuclear scintigraphy (index tests) for the diagnosis and differentiation of subtypes of cardiac amyloidosis.

**Methods and results:**

MEDLINE and Embase electronic databases were searched for studies evaluating the diagnostic performance of CMR or nuclear scintigraphy in detecting cardiac amyloidosis and subsequently in differentiating transthyretin amyloidosis (ATTR) from immunoglobulin light‐chain (AL) amyloidosis. In this meta‐analysis, histopathological examination of tissue from endomyocardial biopsy (EMB) or extra‐cardiac organs were reference standards. Pooled sensitivity, specificity, positive likelihood ratio, and negative likelihood ratio were calculated, and a random effects meta‐analysis was used to estimate diagnostic odds ratios. Methodological quality was assessed using a validated instrument. Of the 2947 studies identified, 27 met the criteria for inclusion. Sensitivity and specificity of CMR in diagnosing cardiac amyloidosis was 85.7% and 92.0% against EMB reference and 78.9% and 93.9% with any organ histology reference. Corresponding sensitivity and specificity of nuclear scintigraphy was 88.4% and 87.2% against EMB reference and 82.0% and 98.8% with histology from any organ. CMR was unable to reliably differentiate ATTR from AL amyloidosis (sensitivity 28.1–99.0% and specificity 11.0–60.0%). Sensitivity and specificity of nuclear scintigraphy in the differentiation of ATTR from AL amyloidosis ranged from 90.9% to 91.5% and from 88.6% to 97.1%. Pooled negative likelihood ratio and positive likelihood ratio for scintigraphy in this setting were 0.1 and 8, with EMB reference standard. Study quality assessed by QUADAS‐2 was generally poor with evidence of bias.

**Conclusions:**

Cardiac magnetic resonance is a useful test for diagnosing cardiac amyloidosis but is not reliable in further classifying the disease. Nuclear scintigraphy offers strong diagnostic performance in both the detection of cardiac amyloidosis and differentiating ATTR from AL amyloidosis. Our findings support the use of both imaging modalities in a non‐invasive diagnostic algorithm that also tests for the presence of monoclonal protein.

## 1 Introduction

Amyloidosis is frequently misdiagnosed and delayed in its recognition, in part due to a perception that it is rare and difficult to diagnose.^1^ However, screening studies using non‐invasive imaging in selected populations^2^ have detected TTR amyloid deposition in 13% of patients hospitalised for heart failure with preserved ejection fraction^3^; 16% of patients undergoing transcatheter aortic valve implantation for severe aortic stenosis^4^; and 5% of patients with presumed hypertrophic cardiomyopathy.^5^ Taken together, these findings suggest a significant underdiagnosis of transthyretin amyloidosis (ATTR) across a range of heart disease phenotypes.

Immunoglobulin light‐chain (AL) amyloidosis and ATTR account for 90% of all systemic forms,^6^ and their timely differentiation is critical. Left untreated, AL amyloidosis is associated with a very poor prognosis and < 1 year median survival in the presence of cardiomyopathy^7^, ^8^; however, chemotherapy directed at the underlying plasma cell dyscrasia can prolong median survival to beyond 3 years.^9^ Transthyretin amyloid cardiomyopathy (ATTR‐CM), caused by deposition of misfolded TTR protein in the heart, progresses slower than AL but is also fatal. Infiltration of the myocardium and conduction system leads to heart failure and death,^10^ but in contrast to AL amyloidosis, no disease‐modifying treatments are currently licensed for use in Europe. Observational studies in patients with ATTR‐CM suggest that median survival, in the absence of any disease‐modifying treatment, varies between 3 and 5 years.^11^, ^12^, ^13^ Tafamidis, a TTR stabiliser, is the only medicine that has been evaluated in a completed Phase 3 trial in patients with ATTR‐CM.^14^ The Transthyretin Amyloidosis Cardiomyopathy Clinical Trial (ATTR‐ACT) showed a reduction in the incidence all‐cause mortality and cardiovascular‐related hospitalisations with tafamidis as compared with placebo among patients with hereditary or wild‐type ATTR‐CM.^14^ A further Phase 3 trial of a small‐interfering RNA therapeutic, revusiran, was terminated early due to a mortality imbalance.^15^ Other small‐interfering RNAs, or the use of antisense oligonucleotides, could offer alternative therapeutic approaches that remain to be investigated in ATTR‐CM.^16^

Contrast enhanced cardiac magnetic resonance (CMR) is useful in establishing the aetiology of heart failure^17^ and can be suggestive of specific causes of cardiomyopathy by virtue of its ability to detect expansion of the myocardial interstitium caused by inflammation, fibrosis, or extracellular deposition of amyloid proteins.^18^ CMR is thought to offer a greater diagnostic value than echocardiography in detecting cardiac amyloidosis^19^ but may be limited in its ability to distinguish the two predominant subtypes. When CMR is combined with nuclear scintigraphy and a comprehensive screen for monoclonal protein, the need for a tissue diagnosis is obviated in the majority of patients with suspected cardiac amyloidosis.

The diagnostic performance of CMR and nuclear scintigraphy has been reported in individual studies and in single‐modality meta‐analyses.^20^, ^21^ However, the reference histopathological tests have included a combination of cardiac and extra‐cardiac tissue samples, and the results of different techniques using the same imaging modality have been pooled in analyses. The aim of this study was to conduct a comprehensive overview of the accuracy and precision of these modalities and to systematically assess the diagnostic performance of CMR and nuclear scintigraphy for the detection of cardiac amyloidosis and for the subsequent differentiation of ATTR from AL amyloidosis, with histopathology from the heart or other organs considered separately as reference standards.

## 2 Methods

### 2.1 Search strategy and selection criteria

A systematic search of English‐language literature in MEDLINE and Embase databases was performed for relevant publications from inception to 7 November 2018. Our prospective protocol included Medical Subject Headings terms and keywords related to a diagnosis of ATTR‐CM and cardiac amyloidosis, the reference diagnostic test (endomyocardial or extra‐cardiac biopsy), and index imaging tests (CMR and nuclear scintigraphy). Details of the search strategy are shown in the Supporting Information, *Appendix S1*. We also searched the Cochrane Library for any additional data published before November 2018. The database search was supplemented by examining reference lists of included studies and review articles.^20^, ^21^, ^22^

Our inclusion criteria specified studies evaluating the performance of an index test for ATTR‐CM or cardiac amyloidosis against a reference test. Investigations considered as an appropriate reference test were histological confirmation in tissue taken at endomyocardial biopsy (EMB) or from an extra‐cardiac organ.^23^ CMR and nuclear scintigraphy were selected as the index imaging investigations to be evaluated by virtue of their inclusion in a consensus diagnostic algorithm^2^; echocardiography also features but was excluded from our analyses because of the difficulty in pooling data across highly heterogenous techniques and parameters measured. Only studies reporting on ≥5 patients with ATTR‐CM or cardiac amyloidosis were considered. Finally, those that did not report data in a format that permitted the calculation of sensitivity and specificity, and therefore likelihood ratios, were excluded.

### 2.2 Data extraction and quality assessment

Two independent reviewers (J. B. and M. L.) examined the electronic searches and extracted information on study characteristics, quality, and test results. Discrepancies were solved by a discussion between J. B. and M. L., or with P. E. Information was obtained on year of publication, country of origin, sample size, patient demographics, index test, and reference test. Methodological quality of included studies was independently assessed using the QUADAS tool.^24^ This systematic review was conducted according to the protocol registered with PROSPERO (registration no. CRD42018118065) and in accordance with PRISMA guidance.^25^

### 2.3 Statistical analysis

Positive likelihood ratio (PLR) and negative likelihood ratio (NLR) were calculated for each study. The likelihood ratio expresses the magnitude by which the probability of disease is modified in a given patient by the outcome of an index test. A PLR of ≥10 and an NLR of ≤0.1 were considered to provide convincing diagnostic evidence, whereas those between 5 and 10, and 0.1 and 0.2 give strong diagnostic evidence.^26^, ^27^ In light of the importance of avoiding a misdiagnosis of ATTR‐CM in a patient with AL amyloidosis,^2^, ^28^ a very high specificity (≥95%) and high PLR (≥10) were considered the most important performance measures for an index test in the detection of ATTR amyloidosis. In contrast, the performance of a test in an individual in whom cardiac amyloidosis is suspected is best evaluated by the ability of a negative test to rule out cardiac amyloidosis, and therefore, a high sensitivity (≥90%) and low NLR (≤0.1) are the most important measures. Those patients with a positive test result will require additional investigations, including the reference tests used as a benchmark in this meta‐analysis in the case of AL amyloidosis. In the context of detecting cardiac amyloidosis, the results of nuclear scintigraphy scans were considered as positive when reported as Grade ≥ 1 using the Perugini visual score,^29^ and negative when 0. In contrast, when used for differentiating ATTR from AL amyloidosis, positive nuclear scintigraphy scans were defined as a Perugini Grade ≥ 2. These thresholds were used based on a previous study pooling data from 8 centres that showed fewer false negatives when detecting cardiac amyloidosis and fewer false positives in diagnosing ATTR amyloidosis when using this approach.^2^

The pooled diagnostic odds ratio (OR) and 95% confidence interval were estimated using a random effects model,^30^ with an inverse variance method of weighting. The diagnostic OR represents the odds of a test result being positive in an individual with the disease compared with one without the disease. We performed statistical tests of heterogeneity (*I* ^2^) for studies evaluating the performance of nuclear scintigraphy; however, the *I* ^2^ statistic was not calculated across CMR studies because of the variability in cardiac anatomy assessed. Estimates of diagnostic ORs are shown in forest plots according to predefined subgroups based on the reference test (EMB vs. histology from any organ) and the diagnosis (ATTR amyloidosis vs. cardiac amyloidosis). For sensitivity analyses, we planned to assess the effect of excluding studies using extra‐cardiac histopathology as the reference test and those with poor methodological quality as assessed using QUADAS‐2. We did not assess publication bias, because no proven statistical method exists for meta‐analyses of diagnostic accuracy studies.^31^ We used stata version 12 for the analyses.

## 3 Results

**Figure 1:**
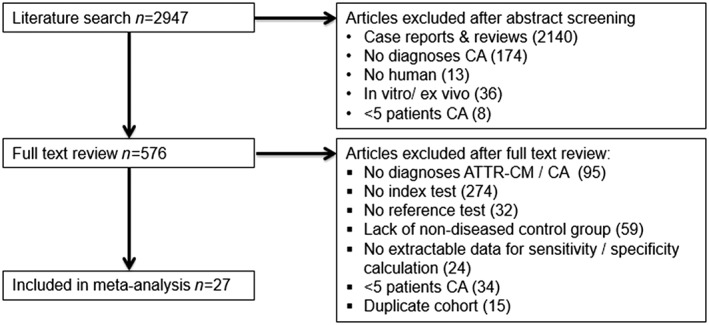
Flow diagram of articles included in the systematic review. ATTR‐CM, transthyretin amyloid cardiomyopathy; CA, cardiac amyloidosis.

Of the 2947 studies, we identified 27 meeting the inclusion criteria, including data on 3493 patients (*Figure* 1). Twenty‐two studies evaluated the performance of CMR in diagnosing either cardiac amyloidosis or ATTR amyloidosis,^19^, ^32^, ^33^, ^34^, ^35^, ^36^, ^37^, ^38^, ^39^, ^40^, ^41^, ^42^, ^43^, ^44^, ^45^, ^46^, ^47^, ^48^, ^49^ with a further five studies assessing the diagnostic performance of nuclear scintigraphy.^2^, ^50^, ^51^, ^52^, ^53^ Seventeen studies assessing the performance of index tests in the diagnosis of cardiac amyloidosis included patients with a range of cardiac conditions, whereas 9 out of 11 studies diagnosing ATTR amyloidosis included patients with ATTR or AL amyloidosis only. Among the four studies using nuclear scintigraphy to differentiate ATTR from AL amyloidosis, three studies specified a monoclonal protein screen in the methodology, the complete results of which were available in only one study.^2^ Among 11 studies using EMB as the reference test, five obtained tissue samples from the right ventricle and one involved biventricular EMB^33^, ^34^, ^35^, ^37^, ^42^, ^49^; the remaining studies did not report on EMB technique. One study reported no complications following EMB in all subjects,^34^ whereas others did not comment on the incidence of complications.

### 3.1 Detecting cardiac amyloidosis using cardiac magnetic resonance or nuclear scintigraphy

Our pre‐specified criteria for diagnostic evidence of cardiac amyloidosis were a high sensitivity (≥90%) and low NLR (≤0.1). *Table* 1 shows the performance of Perugini Grade ≥ 1 nuclear scintigraphy in detecting cardiac amyloidosis. Pooling data across all three tracers evaluated (^99m^Tc‐DPD, ^99m^Tc‐PYP, or ^99m^Tc‐HMDP), nuclear scintigraphy offered strong diagnostic evidence of cardiac amyloidosis but did not fulfil our criteria for convincing test performance against either EMB or any organ histology reference standard. Using an EMB reference, both ^99m^Tc‐DPD and ^99m^Tc‐PYP showed strong diagnostic evidence, with sensitivities of 89.0% and NLRs of 0.1.

**Table 1:** Pooled diagnostic performance in detecting cardiac amyloidosis among patients with clinical suspicion

Parameter	No. of patients	Prevalence, n (%)	Sensitivity (%)	Specificity (%)	PLR	NLR
Grade 1/2/3 nuclear scintigraphy vs. EMB reference						
99mTc‐DPD ≥ 1^2^	244	209 (85.6)	89.0	88.6	8	0.1
99mTc‐PYP ≥ 1^2^	109	100 (91.7)	89.0	77.8	4	0.1
99mTc‐HMDP ≥ 1^2^	21	18 (85.7)	77.8	100	3	0.2
Summary	374	327 (87.4)	88.4	87.2	7	0.1
Grade 1/2/3 nuclear scintigraphy vs. any organ histology						
99mTc‐DPD ≥ 1^2^, ^53^	991	559 (56.4)	81.9	98.8	71	0.2
99mTc‐PYP ≥ 1^2^	193	145 (75.1)	91.7	95.8	22	0.1
99mTc‐HMDP ≥ 1^2^, ^52^	226	129 (57.1)	71.3	100	70	0.3
Summary	1410	833 (59.1)	82.0	98.8	68	0.2
Magnetic resonance vs. EMB reference						
LGE subendocardium^34^, ^35^, ^37^	92	42 (45.7)	85.7	92.0	11	0.2
Magnetic resonance vs. any organ histology						
LGE atria^32^, ^36^	118	39 (33.0)	74.4	91.1	8	0.3
LGE subendocardium^19^, ^34^, ^35^, ^37^	137	71 (51.8)	78.9	93.9	13	0.2

DT, deceleration time; EF, ejection fraction; LA, left atrial; LGE, late gadolinium enhancement; NLR, negative likelihood ratio; OR, odds ratio; PLR, positive likelihood ratio.

Studies assessing the performance of CMR in detecting cardiac amyloidosis used the distribution of late gadolinium enhancement (LGE), thresholds of native T1 values, or extracellular volume as an index test (Supporting Information, *Appendix* *S2*). Patients included in these studies were selected either based on a clinical suspicion of cardiac amyloidosis (definition varied) or a known diagnosis of cardiac amyloidosis with a control cohort. The confirmed diagnoses of patients included in 11 studies assessing the performance of LGE in varying distributions for the detection of cardiac amyloidosis are shown in the Supporting Information, *Appendix* *S3*. After cardiac amyloidosis (*n* = 253, 49%), the most common diagnoses were hypertensive heart disease (*n* = 80, 15%), hypertrophic cardiomyopathy (*n* = 31, 6%), lysosomal storage disease (*n* = 24, 5%), and non‐ischaemic dilated cardiomyopathy (*n* = 22, 4%).

**Figure 2:**
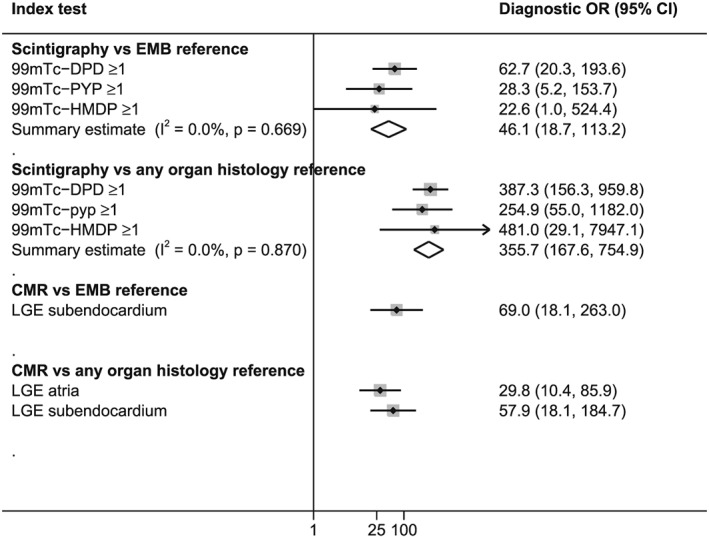
Pooled diagnostic odds ratios for detecting cardiac amyloidosis. LA, left atrium; LGE, late gadolinium enhancement; LV, left ventricle; MR, magnetic resonance; OR, odds ratio; RA, right ventricle; RV, right ventricle.

Subendocardial LGE, assessed in patients with suspected cardiac amyloidosis, was defined as globally distributed in the subendocardium^19^ or in a distribution over the entire subendocardial circumference,^34^, ^35^, ^36^ irrespective of extension into myocardium. The pooled sensitivity of subendocardial LGE in patients with suspected cardiac amyloidosis ranged from 78.9% with histology from any organ as the reference standard to 85.7% with EMB (*Table* 1). NLRs for subendocardial LGE were 0.2 against both reference standard tests, falling just short of our pre‐specified markers of convincing performance. Atrial LGE, also assessed in patients with suspected cardiac amyloidosis, was a less useful measure than subendocardial LGE, as both pooled sensitivity (74.4%) and NLR (0.3) were poorer. Diagnostic ORs in CMR tests for the detection of cardiac amyloidosis are shown in *Figure* 2. An individual with confirmed cardiac amyloidosis on EMB was 69 times more likely to have subendocardial LGE than an individual without cardiac amyloidosis. In comparison, an individual with cardiac amyloidosis was 46 times more likely to have a Perugini Grade of ≥1 nuclear scintigraphy scan than those without cardiac amyloidosis.

Three studies included patients with known systemic amyloidosis or a plasma cell dyscrasia only, and evaluated the use of CMR for the purpose of identifying those with cardiac involvement. Each study assessed the presence of LGE in any distribution that was defined by presence in the subendocardium, myocardium, or a patchy distribution in two studies, and using a semi‐automated technique in another. The presence of LGE in any distribution in this setting performed well against a reference standard of any organ histology, with a pooled sensitivity of 93.0% and an NLR of <0.1 (Supporting Information, *Appendix* *S2*).

Three studies assessed native T1 values for the detection of cardiac amyloidosis, with sensitivities ranging from 74.0% to 84.2% and NLRs from <0.1 to 0.2. We were unable to pool results across these studies due to the use of inconsistent T1 value thresholds. A single study reporting on extracellular volume measurements for diagnosing cardiac amyloidosis against an EMB reference reported a sensitivity and NLR of 95.7% and < 0.1, respectively. A single study evaluated ventricular LGE among patients with suspected cardiac amyloidosis, reporting good performance of LV LGE for its identification (sensitivity of 94.1% and NLR of 0.1).

### 3.2 Differentiating transthyretin amyloidosis from immunoglobulin light‐chain amyloidosis using cardiac magnetic resonance or nuclear scintigraphy

Our pre‐specified criteria for differentiating ATTR from AL amyloidosis were a specificity of ≥95% and PLR of ≥10 (*Table* 2). Nuclear scintigraphy with a Grade of ≥2 using any of ^99m^Tc‐DPD, ^99m^Tc‐PYP, or ^99m^Tc‐HMDP showed a PLR of >10 when evaluated against a reference test of histology from any organ, and specificity varied between 92.2% and 100%. The pooled PLR fell to 8 among nuclear scintigraphy studies using histopathology based on EMB alone. One study using Grade ≥ 2 nuclear scintigraphy with any one of the three tracers, combined with the absence of monoclonal protein in serum and urine immunofixation electrophoresis and serum free light‐chain (sFLA) assay, reported a specificity of 100%, both among 1217 patients with histology from any organ and in 374 patients with EMB sourced histology (Supporting Information, *Appendix* *S4*).^2^

**Table 2:** Pooled diagnostic performance in differentiating ATTR‐CM from AL amyloidosis

Index test	No. of patients	Prevalence, n (%)	Sensitivity (%)	Specificity (%)	PLR	NLR
Grade 2/3 nuclear scintigraphy vs. EMB reference						
99mTc‐DPD ≥ 2^2^, ^51^	262	170 (71.4)	94.7	90.2	10	<0.1
99mTc‐PYP ≥ 2^2^	109	85 (78.0)	87.1	79.2	4	0.2
99mTc‐HMDP ≥ 2^2^	21	14 (66.7)	78.6	100.0	6	0.2
Summary	392	269 (68.6)	91.5	88.6	8	0.1
Grade 2/3 nuclear scintigraphy vs. any organ histology						
99mTc‐DPD ≥ 2^2^, ^50^, ^51^	917	379 (41.3)	94.5	97.2	34	<0.1
99mTc‐PYP ≥ 2^2^	199	122 (61.3)	83.6	92.2	11	0.2
99mTc‐HMDP ≥ 2^2^, ^52^	205	89 (43.4)	85.4	100.0	100	0.1
Summary	1321	590 (44.7)	90.9	97.1	32	0.1
Magnetic resonance vs. any organ histology						
LGE LA^41^, ^43^	143	73 (51.0)	78.1	60.0	2	0.4
LGE RV^41^, ^45^, ^54^	535	377 (70.5)	93.9	34.2	1	0.2
LGE LV^43^, ^44^, ^54^	168	95 (56.5)	99.0	11.0	1	0.1
LGE subendocardium^42^, ^54^	132	64 (48.5)	28.1	48.5	1	1.5
LGE transmural^45^, ^54^	410	314 (76.6)	74.2	56.3	2	0.5

LA, left atrium; LGE, late gadolinium enhancement; LV, left ventricle; MR, magnetic resonance; NLR, negative likelihood ratio; OR, odds ratio; PLR, positive likelihood ratio; RA, right ventricle; RV, right ventricle.

**Figure 3:**
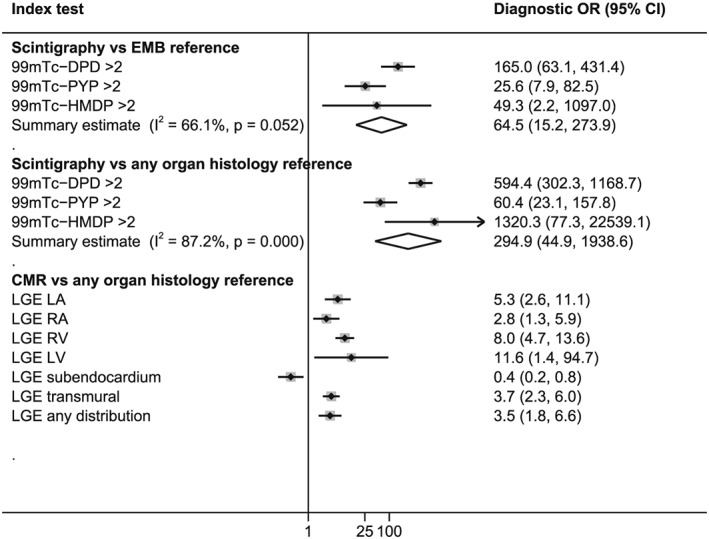
Pooled diagnostic odds ratios for Grade 2/3 scintigraphy and cardiac magnetic resonance in differentiating ATTR from AL cardiac amyloidosis. LA, left atrium; LGE, late gadolinium enhancement; LV, left ventricle; MR, magnetic resonance; OR, odds ratio; RA, right ventricle; RV, right ventricle.

Cardiac magnetic resonance performed poorly in differentiating ATTR from AL amyloidosis; the predefined measures of interest, specificity and PLR, were consistently lower across all CMR index test parameters when compared with corresponding measures for any nuclear scintigraphy test. Studies evaluating the performance of CMR in this setting all used the presence of LGE in varying regions of the heart as the index test (*Figure* 3). Left ventricular LGE was defined by presence in the LV myocardium^43^, ^54^ or any of the 17 LV segments.^44^ One study described subendocardial LGE when there was global subendocardial involvement but no transmural LGE;^54^ another study defined it as global subendocardial LGE irrespective of transmural involvement.^42^ Specificity of CMR in differentiating ATTR from AL amyloidosis across all LGE distributions varied from 11.0% to 60.0%, and PLRs varied from 1 to 2.

### 3.3 Quality assessment

Of the 28 studies meta‐analysed, all scored high risk of bias in the QUADAS‐2 flow and timing domain (Supporting Information, *Appendix S5*). This was because no single study reported on blinding of index and reference test reporting, or the timing of index and reference tests. Included studies performed better in other domains with nine studies scored as high risk of bias in patient selection, and all studies scoring low risk in the other four domains of QAUDAS‐2, including index test and reference test in both risk of bias and applicability areas. Given that no included study scored a low risk of bias across all domains, the planned subgroup analysis excluding studies with high risk of bias was not performed.

## 4 Discussion

Cardiac magnetic resonance provides strong diagnostic evidence for cardiac amyloidosis but is unable to effectively differentiate its subtypes. Nuclear scintigraphy offers comparable performance in diagnosing cardiac amyloidosis but can also reliably differentiate ATTR from AL amyloidosis affecting the heart, when combined with a monoclonal protein screen. The findings of this meta‐analysis suggest that both modalities offer sensitivity >85% for the diagnosis of cardiac amyloidosis among patients with a clinical suspicion of the disease, with low false positive rates. Based on the likelihood ratios reported here, the absence of subendocardial LGE on CMR reduces the pre‐test probability of cardiac amyloidosis by around 30%, and the absence of any cardiac uptake on nuclear scintigraphy by 45%.^55^ It has been suggested that the poor sensitivity and specificity of echocardiography in detecting cardiac amyloidosis may contribute to the substantial misdiagnosis or delay in diagnosing it.^2^, ^56^ Our findings indicate that early use of nuclear scintigraphy among patients with a high index of suspicion for cardiac amyloidosis may offer an opportunity to accelerate diagnosis, prompting earlier referral for specialised assessment and to direct further treatment.

The classical echocardiographic features suggesting cardiac amyloidosis are concentric left ventricular wall thickening, atrial septum thickening, and increased echogenicity of the ventricular septum described as granular sparkling.^23^, ^57^, ^58^ In practice, the disease is phenotypically heterogeneous, and CMR evaluation in cardiac amyloidosis suggests that 42% can have a remodelling pattern different from concentric increase in LV wall thickness, including asymmetric and eccentric remodelling.^10^, ^59^ Furthermore, the sensitivity of granular sparkling in diagnosing cardiac amyloidosis among patients with a clinical suspicion has been reported as <50%.^60^, ^61^ Despite being a valuable modality for investigating heart failure and suggesting the possibility of cardiac amyloidosis, the ability of the classical echocardiographic features to make this diagnosis has been limited by poor specificity and sensitivity.^56^ We did not perform a systematic assessment of echocardiography in diagnosing cardiac amyloidosis due to the significant heterogeneity in technique and parameters measured. However, several novel echocardiogram parameters in isolation or in combination have shown promising diagnostic performance (low NLR and high sensitivity) in individual studies and merit further investigation.^62^, ^63^, ^64^

Nuclear scintigraphy demonstrated good diagnostic performance both in the detection of cardiac amyloidosis using a Perugini grade threshold of ≥1 and in the differentiation of its subtypes using a threshold of ≥2. In this regard, nuclear scintigraphy offers some advantages over CMR in that it is able to detect and differentiate cardiac amyloid; however, CMR yields additional information on ventricular function and morphology, and is able to detect a broader range of cardiomyopathies.^18^ As such, in patients with a high index of suspicion for cardiac amyloidosis, early use of nuclear scintigraphy should be considered as an alternative to CMR imaging. It is critical that any test used to differentiate ATTR from AL amyloidosis has a near 100% specificity, so as not to miss AL amyloidosis requiring urgent chemotherapy. The seminal scintigraphy study included in this meta‐analysis, which pooled international data from 10 amyloidosis centres, highlights the importance of performing a comprehensive monoclonal protein screen (including serum and urine immunofixation electrophoresis and serum free light chain (sFLC) assay). When a negative monoclonal protein screen is combined with Grade 2/3 scintigraphy, specificity improved from 87% to 100% among patients with EMB‐based histopathology. Reporting of this ‘triple screen’ for monoclonal protein, in combination with imaging, was incomplete in the remaining three studies that assessed performance of scintigraphy in differentiating ATTR from AL amyloidosis; therefore, we were unable to add additional data on the combined investigations in our study.

In addition to the requirement for histological demonstration and typing of amyloid among patients with a monoclonal protein, those with low cardiac uptake (Grade 1) on nuclear scintigraphy in the absence of a monoclonal protein also require histological typing of amyloid. This is because of the low specificity of any cardiac uptake (Grade 1, 2, or 3) for a diagnosis of ATTR cardiac amyloid resulting mainly from low‐grade uptake in patients with cardiac AL.^65^ Results from specialist amyloid centres suggest that as many as 19% of patients with ATTR amyloidosis will have a monoclonal protein and 9% will have low cardiac uptake (Grade 1),^2^ demonstrating a clear ongoing need for EMB in some patients. While extra‐cardiac biopsy in clinically suspected amyloidosis can yield a diagnosis in 50% to 80% of patients with AL amyloidosis,^66^ this falls to 35% in patients with wild‐type ATTR‐CM.^67^ Furthermore, because of the potential for false negative results for ATTR amyloidosis with a non‐invasive approach, a histological diagnosis from EMB should be sought in any patient with suspected cardiac amyloidosis where non‐invasive diagnostic criteria are not met.^2^

In this analysis, we pooled the largest set of accuracy and precision data so far, resulting in a four‐fold increase in patient numbers with respect to CMR tests in detecting cardiac amyloidosis.^21^ Furthermore, we were able to categorise histological reference tests according to whether an EMB was performed or not. A previously published meta‐analysis that pooled performance of CMR across five studies reported sensitivity of 85% and specificity of 92% for the presence of any LGE, using a combination of reference tests including clinical features and echocardiography.^21^ Our analyses provide additional information with respect to patient numbers, the use of histological confirmation of diagnosis stratified by organ, and finally on the distribution of LGE in the heart. We were also able to provide a novel summary of the performance of CMR in differentiating ATTR from AL amyloidosis. Our study adds only a modest increase in patient numbers undergoing scintigraphy due to the dominant weighting of one seminal study evaluating this modality.^2^ The addition of data from four relatively small studies to that provided in the seminal scintigraphy study reinforce the importance of combining this test with the triple monoclonal protein screen in order to obtain a specificity of 100% in differentiating ATTR from AL amyloidosis.

Across studies using EMB and any organ histology as a reference test, we recorded consistent values in the important measures of sensitivity and NLR for the detection of cardiac amyloidosis with CMR. In contrast, the specificity and PLR (important measures in differentiating ATTR from AL amyloidosis) fell when studies performing histology on extra‐cardiac biopsies were excluded from our analyses on nuclear scintigraphy. This discrepancy in performance of scintigraphy, when comparing EMB or any organ histology reference standards may relate to a reluctance to perform an EMB in elderly patients with multiple co‐morbidities or in those with more advanced disease. It is possible that the likelihood of a positive scan on nuclear scintigraphy may increase with age or severity of disease.^68^ Therefore, given patients with wild‐type ATTR‐CM present at a more advanced age than those with hereditary ATTR‐CM, both older and more advanced patients who are considered unfit to undergo EMB could be more likely to have a positive scan.

Large historical case series suggest that rates of complications from EMB vary between 0% and 3%^69^, ^70^, ^71^; however, the rate of major complications including ventricular perforation, pericardial tamponade, and embolisation have clearly improved over time. Contemporary series suggest that rates as low as 0% and 0.3% may be achievable with high levels of experience for left and right ventricular EMB, respectively.^70^ As demonstrated in the included studies in this meta‐analysis, right ventricular biopsy is the more commonly used technique.^72^ The largest head‐to‐head comparison in unselected patients who underwent biventricular EMB found that 96% of left ventricular and 71% of right ventricular showed diagnostic histopathological findings; however, results were comparable when echocardiography was used to detect the most affected chamber.^70^ In contrast, the role of CMR‐guided targeting of areas of LGE is more uncertain as there are conflicting reports on its impact on diagnostic yield.^73^, ^74^

There were many sources of potential heterogeneity among included studies. While studies evaluating the use of imaging modalities in diagnosing ATTR amyloidosis were confined to a homogenous group of patients with either ATTR or AL amyloidosis, CMR studies in the detection of cardiac amyloidosis included patients with a wide range of cardiac conditions. All studies contributing data to our analyses reported on clinically relevant populations undergoing CMR for suspected cardiac amyloidosis; however, the underlying diagnosis of included patients will influence the presence and distribution of LGE. We excluded studies evaluating the performance of tests in healthy individuals to ensure that our analyses reflected clinical practice; however, it is possible that the performance of tests will differ depending on the non‐diseased control population studied. Differences in the imaging protocols between studies, and in the interpretation of CMR, may also contribute to heterogeneity. Our findings may be biased by the expert nature of centres contributing data that may have imaging experience that is not widely applicable, particularly with respect to CMR. This bias could lead to an overestimation of the diagnostic performance of index tests. The width of confidence intervals in our pooled diagnostic ORs was large and may relate to the limited number of studies and between study heterogeneity that widens confidence intervals in a random effects meta‐analysis.

The findings of this meta‐analysis provide strong evidence for the early use of nuclear scintigraphy among patients with a strong clinical suspicion of cardiac amyloidosis. We show that nuclear scintigraphy performs consistently well in diagnosing cardiac amyloidosis and differentiating its subtypes. Importantly, additional investigations that combine scintigraphy with a triple screen for monoclonal protein are required to reach the necessary near 100% specificity for a diagnosis of ATTR cardiac amyloidosis. CMR also offers strong diagnostic evidence for cardiac amyloidosis, and the sensitivity of subendocardial LGE is superior compared with previous reports assessing echocardiography for this purpose. CMR is not a good test for differentiating ATTR‐CM from AL amyloidosis. Our findings should prompt health care services that interact with patients with suspected cardiac amyloidosis to consider incorporating nuclear scintigraphy into their practice.

## Conflict of interest

J.B. and M.F.L. are full time employees of Pfizer and hold stock and/or stock options. M.L. and P.E. are paid consultants to Pfizer, including in connection with the development of this manuscript. P.E. has also received educational grants from Pfizer and consultancy fees from Alnylam.

## Funding

This study was sponsored by Pfizer.

## Supporting information


**Appendix S1.** Search terms.
**Appendix S2.** Diagnostic performance for the detection of cardiac amyloidosis.
**Appendix S3.** Description of population and late gadolinium enhacement in studies evaluating CMR for the detection of CA.
**Appendix S4.** Diagnostic performance for detecting ATTR‐CM.
**Appendix S5.** Methodological quality of included studies using the QUADAS‐2 tool.
