# Supplementary material for: Diagnostic performance of imaging investigations in detecting and differentiating cardiac amyloidosis: a systematic review and meta‐analysis
Source: ESC Heart Fail. 2019 Sep 5;6(5):1041–51. doi: 10.1002/ehf2.12511 (PMC6816075; doi:10.1002/ehf2.12511)
Supplement: Supplementary file 1 — Appendix S1. Search terms. Appendix S2. Diagnostic performance for the detection of cardiac amyloidosis. Appendix S3. Description of population and late gadolinium enhacement in studies evaluating CMR for the detection of CA. Appendix S4. Diagnostic performance for detecting ATTR‐CM. Appendix S5. Methodological quality of included studies using the QUADAS‐2 tool. [file EHF2-6-1041-s001.doc]

**Diagnostic Performance of Imaging Investigations in Detecting and Differentiating Cardiac Amyloidosis: a Systematic Review and Meta-analysis**

**APPENDIX 1. Search terms**

ATTR and CA

1. Transthyretin*.ti.ab
2. Transthyretin amyloid*.ti.ab
3. Cardiac amyloid*.ti.ab
4. Systemic amyloid*.ti.ab
5. ATTR.ti.ab
6. TTR.ti.ab
7. Light chain amyloidosis.ti.ab
8. AL amyloidosis.ti.ab
9. OR/ 1-8

Biopsy

1. Biopsy*.ti.ab
2. Histopathology*.ti.ab
3. Histology*.ti.ab

Bone scan

1. Scintigraphy*.ti.ab
2. Technetium*.ti.ab
3. Nuclear imaging*.ti.ab
4. Radionuclide*.ti.ab

CMR

1. Magnetic resonance*.ti.ab
2. CMR.ti.ab
3. (Cardiac AND MR).ti.ab

SEARCH STRATEGY

ATTR AND (Biopsy OR Bone scan OR CMR)

**APPENDIX 2. Diagnostic performance for the detection of cardiac amyloidosis**

| **Source** | **No. patients** | **Study popn** | **Reference test** | **Index test** | **True positive** | **False negative** | **True negative** | **False positive** | **Sensitivity (%)** | **Specificity (%)** | **PLR** | **NLR** |
| --- | --- | --- | --- | --- | --- | --- | --- | --- | --- | --- | --- | --- |
| **Nuclear Scintigraphy** | | | | | | | | | | | | |
| Capelli et al^1^ 2017 | 85 | Suspected Ca | Histology from any organ | ^99m^Tc-HMDP ≥1 | 41 | 24 | 20 | 0 | 63 | 100 | NA | 0.4 |
| Cariou et al^2^ 2017 | 114 | LVH | Histology from any organ | ^99m^Tc-DPD ≥1 | 31 | 19 | 64 | 0 | 62 | 100 | NA | 0.4 |
| Gillmore et al^3^ 2016 | 1076 | Suspected CA | Histology from any organ | ^99m^Tc-DPD ≥1  ^99m^Tc-PYP ≥1  ^99m^Tc-HMDP ≥1 | 427  133  51 | 82  12  13 | 363  46  77 | 5  2  0 | 84  92  80 | 99  96  100 | 62  22  NA | 0.2  0.1  0.2 |
| Gillmore et al^3^ 2016 | 374 | Suspected CA | EMB | ^99m^Tc-DPD ≥1  ^99m^Tc-PYP ≥1  ^99m^Tc-HMDP ≥1 | 186  89  14 | 23  11  4 | 31  7  3 | 4  2  0 | 89  89  78 | 89  78  100 | 8  4  NA | 0.1  0.1  0.2 |
| **Cardiac magnetic resonance** | | | | | | | | | | | | |
| Mongeon et al^4^ 2012 | 38 | CA, LVH | EMB | LGE atria  LGE LV  LGE RV | 12  16  12 | 5  1  5 | 20  17  20 | 0  3  0 | 71  94  71 | 100  85  100 | NA  6  NA | 0.3  0.1  0.3 |
| White et al^5^ 2014 | 25 | Suspected CA | EMB | LGE LV - extensive (>50%) | 14 | 1 | 7 | 3 | 93 | 70 | 3 | 0.1 |
| Vogelsberg et al^6^ 2008 | 33 | Suspected CA | EMB | LGE subendocardium | 12 | 3 | 17 | 1 | 80 | 94 | 14 | 0.2 |
| Austin et al^7^ 2009 | 38 | Suspected CA | EMB | LGE subendocardium | 15 | 2 | 19 | 2 | 88 | 90 | 9 | 0.1 |
| Austin et al^7^ 2009 | 47 | Suspected CA | Histology from any organ | LGE subendocardium | 19 | 6 | 19 | 3 | 76 | 86 | 6 | 0.3 |
| Kwong et al^8^ 2015 | 81 | CA, HTN, DCM | EMB | LGE atria | 17 | 5 | 52 | 7 | 77 | 88 | 7 | 0.3 |
| Baroni et al^9^ 2018 | 21 | Suspected CA | EMB | LGE subendocardium | 9 | 1 | 10 | 1 | 90 | 91 | 10 | 0.1 |
| Maceira et al^10^ 2005 | 45 | CA, HTN | Histology from any organ | LGE subendocardium | 20 | 9 | 16 | 0 | 69 | 100 | NA | 0.3 |
| Bhatti et al^11^ 2016 | 42 | Plasma cell dyscrasia | EMB | LGE (any) | 22 | 0 | 16 | 4 | 100 | 80 | 5 | <0.1 |
| Ruberg et al^12^ 2009 | 28 | Systemic amyloidosis | Histology from any organ | LGE (any) | 18 | 3 | 6 | 1 | 86 | 86 | 6 | 0.2 |
| Karamitsos et al^13^ 2013 | 42 | Systemic amyloisosis | Histology from any organ | LGE (any) | 26 | 2 | 14 | 0 | 93 | 100 | NA | <0.1 |
| Williams et al^14^ 2017 | 83 | CA, HCM, AFD | Histology from any organ | Threshold total LGE burden | NA | NA | NA | NA | 98 | 45 | 2 | <0.1 |
| Da Nam et al^15^ 2018 | 76 | CA, HCM | EMB | Native T1 value >1130  Extracellular vol >40 | NA  NA | NA  NA | NA  NA | NA  NA | 76.1  95.7 | 83.3  93.3 | 5  14 | 0.3  <0.1 |
| Fontana et al^16^ 2014 | 236 | CA, HCM | Histology from any organ | Native T1 value >1065 | NA | NA | NA | NA | 74.0 | 85.0 | 5 | 0.3 |
| Hosch et al^17^ 2007 | 29 | Systemic amyloidosis | Histology from any organ | Native T1 value >1273 | 16 | 3 | 8 | 1 | 84.2 | 88.9 | 8 | 0.2 |

| **Source** | **Inclusions** | **Final diagnoses** | **Index test** | **LGE distribution / pattern description** |
| --- | --- | --- | --- | --- |
| Mongeon et al^4^ 2012 | Assessment of infiltrative heart disease (n=38) | CA (17)  Hypertensive cardiomyopathy (20)  Hypertrophic cardiomyopathy (1)  Lysosomal storage disease (1)  Ventricular arrhythmias (1)  Cardiomyopathy undefined aetiology (9) | LGE atria  LGE LV  LGE RV | Presence or absence (qualitative assessment) in each of 17 myocardial segments |
| White et al^5^ 2014 | Suspected CA | CA (15)  Myocarditis (1)  Normal (4)  Non-specific fibrosis (5) | LGE - extensive | LGE defined as >50% LV myocardium (not in typical coronary artery distribution) had T1 shorter than that of blood |
| Vogelsberg et al^6^ 2008 | Suspected CA | CA (15)  Myocarditis (6)  Secondary LVH (7)  HCM (4)  Unclear (1) | LGE subendocardium | LGE defined as T1 shorter than that of blood over entire subendocardial surface. Short axes divided into 12 circumferential segments and LGE analysed in outer, middle and inner thirds. |
| Austin et al^7^ 2009 | Suspected CA (n=47) | CA (25)  Glycogen storage disease (4)  HCM (7)  Non-specific fibrosis (9)  Myocarditis (1)  Unknown (1) | LGE subendocardium | LGE in circumferential pattern involving entire subendocardium. LGE measure not reported. |
| Kwong et al^8^ 2015 | Known CA or clinical referral for CMR (n=81) | CA (22)  HTN (37)  NIDCM (22) | LGE atria | Presence or absence (qualitative assessment) in 9 segment model of LA |
| Baroni et al^9^ 2018 | Suspected CA (n=42) | CA (14)  Unknown (28) | LGE subendocardium | Presence or absence (qualitative assessment), LGE subendocardium defined as global subendocardial enhancement with or without transmural enhancement |
| Maceira et al^10^ 2005 | Known CA and hypertensive controls | CA (29)  HTN (16) | LGE subendocardium | 2 regions specified: 1, whole subendocardium (inner third myocardium); 2, whole subepicardium (outer third myocardium). LGE defined quantitatively using blood pool and subendocardial enhancement. |
| Bhatti et al^11^ 2016 | Suspected CA and plasma cell dyscrasia (n=42) | CA (22)  Unknown (20) | LGE-various patterns | LGE defined as presence of LGE in circumferential pattern involving entire subendocardium extending to various degree into myocardium, diffuse myocardial hyper enhancement without nulling, or patchy pattern of hyperenhancement on delayed post contrast sequences. |
| Ruberg et al^12^ 2009 | Suspected CA | CA (21)  Systemic amyloidosis without cardiac involvement (7) | LGE (any) | Presence LGE determined using semiautomated technique using signal intensity threshold above that of myocardium that demonstrated lowest signal intensity (effectively nulled). |
| Karamitsos et al^13^ 2013 | Suspected CA | CA (28)  Systemic amyloidosis without cardiac involvement (14) | LGE (any) | LGE defined as patchy or circumferential subendocardial extending to various degree into myocardium. |
| Williams et al^14^ 2017 | Known CA and controls with HCM and Fabry’s disease | CA (45)  HCM (19)  AFD (19) | Threshold total LGE Burden | Presence LGE determined using semiautomated technique using signal intensity threshold above that of myocardium that demonstrated lowest signal intensity (effectively nulled). LGE analysis performed in 16 myocardial segments. |

**APPENDIX 3. Description of population and late gadolinium enhacement in studies evaluating CMR for the detection of CA**

**APPENDIX 4. Diagnostic performance for detecting ATTR-CM**

| **Source** | **No. patients** | **Study popn** | **Reference test** | **Index test** | **True positive** | | **False negative** | **True negative** | **False positive** | **Sensitivity (%)** | **Specificity (%)** | **PLR** | **NLR** |
| --- | --- | --- | --- | --- | --- | --- | --- | --- | --- | --- | --- | --- | --- |
| **Nuclear Scintigraphy** | | | | | | | | | | | | | |
| Capelli et al^1^ 2017 | 65 | ATTR, AL | Histology from any organ | ^99m^Tc-HMDP ≥2 | 36 | | 3 | 26 | 0 | 92 | 100 | NA | <0.1 |
| Gillmore et al^3^ 2016 | 244 | Suspected CA | EMB | ^99m^Tc-DPD ≥2  ^99m^Tc-PYP ≥2  ^99m^Tc-HMDP ≥2 | 153  74  11 | | 9  11  3 | 72  19  7 | 9  5  0 | 95  87  79 | 90  79  100 | 10  4  NA | 0.1  0.2  0.2 |
| Gillmore et al^3^ 2016 | 1498 | Suspected CA | Histology from any organ | ^99m^Tc-DPD ≥2  ^99m^Tc-PYP ≥2  ^99m^Tc-HMDP ≥2 | 337  102  40 | | 21  20  10 | 505  71  90 | 14  6  0 | 94  84  80 | 82  92  100 | 6  11  NA | 0.1  0.2  0.1 |
| Haro-del Morral et al^18^ 2012 | 19 | ATTR, AL | EMB | ^99m^Tc-DPD ≥2 | 8 | | 0 | 11 | 0 | 100 | 100 | NA | <0.1 |
| Moore et al^19^ 2017 | 21 | ATTR, AL | Histology from any organ | ^99m^Tc-DPD ≥2 | 13 | | 0 | 7 | 1 | 100 | 88 | NA | <0.1 |
| **Cardiac magnetic resonance** | | | | | | | | | | | | | |
| de Gregorio et al^20^ 2016 | 32 | ATTR, HCM | Histology from any organ or scintigraphy | LGE LV  LGE LA | | 10  9 | 0  1 | 3  8 | 5  0 | 100  90 | 38  100 | 2  NA | <0.1  0.1 |
| Dungu et al^21^ 2014 | 97 | ATTR, AL | Histology from any organ | LGE atria  LGE LV  LGE RV  LGE subendocardium  LGE transmural | | 47  51  51  6  46 | 4  0  0  45  5 | 12  1  13  28  29 | 34  45  33  18  17 | 92  100  100  12  90 | 26  2  28  61  63 | 1  1  1  0  2 | 0.3  <0.1  <0.1  1.5  0.2 |
| Fontana et al^22^ 2015 | 250 | ATTR, AL | Histology from any organ or scintigraphy | LGE (any) | | 114 | 17 | 37 | 72 | 87 | 34 | 1 | 0.4 |
| Kristen et al^23^ 2015 | 125 | ATTR, AL | Histology from any organ | LGE LA  LGE RA  LGE RV | | 48  46  51 | 15  17  12 | 34  31  30 | 28  31  32 | 76  73  81 | 55  50  48 | 2  1  2 | 0.4  0.5  0.4 |
| Martinez-Naharro et al^24^ 2017 | 313 | ATTR, AL | Histology from any organ or scintigraphy | LGE RV  LGE transmural | | 187  252 | 76  11 | 25  11 | 25  39 | 71  96 | 50  22 | 1  1 | 0.6  0.2 |
| Syed et al^25^ 2010 | 35 | ATTR, AL | EMB | LGE subendocard | | 12 | 1 | 5 | 17 | 92 | 23 | 1 | 0.3 |
| Ternacle et al^26^ 2016 | 53 | ATTR, AL | Histology from any organ | LGE LV | | 33 | 1 | 4 | 15 | 97 | 21 | 1 | 0.1 |

**APPENDIX 5. Methodological quality of included studies using the QUADAS-2 tool**

| Study | Risk of bias | | | | Applicability concerns | | |
| --- | --- | --- | --- | --- | --- | --- | --- |
|  | Patient selection | Index test | Reference standard | Flow and timing | Patient selection | Index test | Reference standard |
| Mongeon et al^4^ 2012 | L | L | L | H | L | L | L |
| White et al^5^ 2014 | H | L | L | H | L | L | L |
| Vogelsberg et al^6^ 2008 | H | L | L | H | L | L | L |
| Austin et al^7^ 2009 | H | L | L | H | L | L | L |
| Austin et al^7^ 2009 | H | L | L | H | L | L | L |
| Kwong et al^8^ 2015 | L | L | L | H | L | L | L |
| Baroni et al^9^ 2018 | H | L | L | H | L | L | L |
| Maceira et al^10^ 2005 | L | L | L | H | L | L | L |
| Bhatti et al^11^ 2016 | H | L | L | H | L | L | L |
| Ruberg et al^12^ 2009 | L | L | L | H | L | L | L |
| Karamitsos et al^13^ 2013 | L | L | L | H | L | L | L |
| Williams et al^14^ 2017 | L | L | L | H | L | L | L |
| Capelli et al^1^ 2017 | L | L | L | H | L | L | L |
| Gillmore et al^3^ 2016 | L | L | L | H | L | L | L |
| Gillmore et al^3^ 2016 | L | L | L | H | L | L | L |
| Haro-del Morral et al^18^ 2012 | L | L | L | H | L | L | L |
| Moore et al^19^ 2017 | L | L | L | H | L | L | L |
| de Gregorio et al^20^ 2016 | L | L | L | H | L | L | L |
| Damy et al^27^ 2015 | L | L | L | H | H | L | L |
| Dungu et al^21^ 2014 | L | L | L | H | L | L | L |
| Fontana et al^22^ 2015 | L | L | L | H | L | L | L |
| Kristen et al^23^ 2015 | L | L | L | H | L | L | L |
| Martinez-Naharro et al^24^ 2017 | L | L | L | H | L | L | L |
| Syed et al^25^ 2010 | L | L | L | H | L | L | L |
| Ternacle et al^26^ 2016 | L | L | L | H | L | L | L |
| Cariou et al^2^ 2017 | H | L | L | H | L | L | L |

**REFERENCES**

1. Cappelli F, Gallini C, Di Mario C, Costanzo EN, Vaggelli L, Tutino F, Ciaccio A, Bartolini S, Angelotti P, Frusconi S. Accuracy of 99mTc-Hydroxymethylene diphosphonate scintigraphy for diagnosis of transthyretin cardiac amyloidosis. Journal of Nuclear Cardiology 2017:1-8.

2. Cariou E, Bennani Smires Y, Victor G, Robin G, Ribes D, Pascal P, Petermann A, Fournier P, Faguer S, Roncalli J. Diagnostic score for the detection of cardiac amyloidosis in patients with left ventricular hypertrophy and impact on prognosis. Amyloid 2017;**24**(2):101-109.

3. Gillmore JD, Maurer MS, Falk RH, Merlini G, Damy T, Dispenzieri A, Wechalekar AD, Berk JL, Quarta CC, Grogan M. Nonbiopsy Diagnosis of Cardiac Transthyretin AmyloidosisCLINICAL PERSPECTIVE. Circulation 2016;**133**(24):2404-2412.

4. Mongeon F-P, Jerosch-Herold M, Coelho-Filho OR, Blankstein R, Falk RH, Kwong RY. Quantification of extracellular matrix expansion by CMR in infiltrative heart disease. JACC: Cardiovascular Imaging 2012;**5**(9):897-907.

5. White JA, Kim HW, Shah D, Fine N, Kim K-Y, Wendell DC, Al-Jaroudi W, Parker M, Patel M, Gwadry-Sridhar F. CMR imaging with rapid visual T1 assessment predicts mortality in patients suspected of cardiac amyloidosis. JACC: Cardiovascular Imaging 2014;**7**(2):143-156.

6. Vogelsberg H, Mahrholdt H, Deluigi CC, Yilmaz A, Kispert EM, Greulich S, Klingel K, Kandolf R, Sechtem U. Cardiovascular magnetic resonance in clinically suspected cardiac amyloidosis: noninvasive imaging compared to endomyocardial biopsy. Journal of the American College of Cardiology 2008;**51**(10):1022-1030.

7. Austin BA, Tang WW, Rodriguez ER, Tan C, Flamm SD, Taylor DO, Starling RC, Desai MY. Delayed hyper-enhancement magnetic resonance imaging provides incremental diagnostic and prognostic utility in suspected cardiac amyloidosis. JACC: Cardiovascular Imaging 2009;**2**(12):1369-1377.

8. Kwong RY, Heydari B, Abbasi S, Steel K, Al-Mallah M, Wu H, Falk RH. Characterization of cardiac amyloidosis by atrial late gadolinium enhancement using contrast-enhanced cardiac magnetic resonance imaging and correlation with left atrial conduit and contractile function. American Journal of Cardiology 2015;**116**(4):622-629.

9. Baroni M, Nava S, Quattrocchi G, Milazzo A, Giannattasio C, Roghi A, Pedrotti P. Role of cardiovascular magnetic resonance in suspected cardiac amyloidosis: late gadolinium enhancement pattern as mortality predictor. Netherlands Heart Journal 2018;**26**(1):34-40.

10. Maceira AM, Joshi J, Prasad SK, Moon JC, Perugini E, Harding I, Sheppard MN, Poole-Wilson PA, Hawkins PN, Pennell DJ. Cardiovascular magnetic resonance in cardiac amyloidosis. Circulation 2005;**111**(2):186-193.

11. Bhatti S, Watts E, Syed F, Vallurupalli S, Pandey T, Jambekar K, Mazur W, Hakeem A. Clinical and prognostic utility of cardiovascular magnetic resonance imaging in myeloma patients with suspected cardiac amyloidosis. European Heart Journal-Cardiovascular Imaging 2016;**17**(9):970-977.

12. Ruberg FL, Appelbaum E, Davidoff R, Ozonoff A, Kissinger KV, Harrigan C, Skinner M, Manning WJ. Diagnostic and prognostic utility of cardiovascular magnetic resonance imaging in light-chain cardiac amyloidosis. The American journal of cardiology 2009;**103**(4):544-549.

13. Karamitsos TD, Piechnik SK, Banypersad SM, Fontana M, Ntusi NB, Ferreira VM, Whelan CJ, Myerson SG, Robson MD, Hawkins PN. Noncontrast T1 mapping for the diagnosis of cardiac amyloidosis. JACC: Cardiovascular Imaging 2013;**6**(4):488-497.

14. Williams LK, Forero JF, Popovic ZB, Phelan D, Delgado D, Rakowski H, Wintersperger BJ, Thavendiranathan P. Patterns of CMR measured longitudinal strain and its association with late gadolinium enhancement in patients with cardiac amyloidosis and its mimics. Journal of Cardiovascular Magnetic Resonance 2017;**19**(1):61.

15. Da Nam B, Kim SM, Jung HN, Kim Y, Choe YH. Comparison of quantitative imaging parameters using cardiovascular magnetic resonance between cardiac amyloidosis and hypertrophic cardiomyopathy: inversion time scout versus T1 mapping. The international journal of cardiovascular imaging 2018:1-9.

16. Fontana M, Banypersad SM, Treibel TA, Maestrini V, Sado DM, White SK, Pica S, Castelletti S, Piechnik SK, Robson MD. Native T1 mapping in transthyretin amyloidosis. JACC: Cardiovascular Imaging 2014;**7**(2):157-165.

17. Hosch W, Bock M, Libicher M, Ley S, Hegenbart U, Dengler TJ, Katus HA, Kauczor H-U, Kauffmann GW, Kristen AV. MR-relaxometry of myocardial tissue: significant elevation of T1 and T2 relaxation times in cardiac amyloidosis. Investigative radiology 2007;**42**(9):636-642.

18. De Haro-Del Moral FJ, Sánchez-Lajusticia A, Gómez-Bueno M, García-Pavía P, Salas-Antón C, Segovia-Cubero J. Role of cardiac scintigraphy with 99mTc-DPD in the differentiation of cardiac amyloidosis subtype. Revista Española de Cardiología (English Edition) 2012;**65**(5):440-446.

19. Moore PT, Burrage MK, Mackenzie E, Law WP, Korczyk D, Mollee P. The utility of 99mTc-DPD scintigraphy in the diagnosis of cardiac amyloidosis: an Australian experience. Heart, Lung and Circulation 2017;**26**(11):1183-1190.

20. de Gregorio C, Dattilo G, Casale M, Terrizzi A, Donato R, Di Bella G. Left Atrial Morphology, Size and Function in Patients With Transthyretin Cardiac Amyloidosis and Primary Hypertrophic Cardiomyopathy–Comparative Strain Imaging Study–. Circulation Journal 2016;**80**(8):1830-1837.

21. Dungu JN, Valencia O, Pinney JH, Gibbs SD, Rowczenio D, Gilbertson JA, Lachmann HJ, Wechalekar A, Gillmore JD, Whelan CJ. CMR-based differentiation of AL and ATTR cardiac amyloidosis. JACC: Cardiovascular Imaging 2014;**7**(2):133-142.

22. Fontana M, Pica S, Reant P, Abdel-Gadir A, Treibel TA, Banypersad SM, Maestrini V, Barcella W, Rosmini S, Bulluck H. Prognostic value of late gadolinium enhancement cardiovascular magnetic resonance in cardiac amyloidosis. Circulation 2015;**132**(16):1570-1579.

23. Kristen AV, aus dem Siepen F, Scherer K, Kammerer R, Andre F, Buss SJ, Bauer R, Lehrke S, Voss A, Giannitsis E. Comparison of different types of cardiac amyloidosis by cardiac magnetic resonance imaging. Amyloid 2015;**22**(2):132-141.

24. Martinez-Naharro A, Treibel TA, Abdel-Gadir A, Bulluck H, Zumbo G, Knight DS, Kotecha T, Francis R, Hutt DF, Rezk T. Magnetic resonance in transthyretin cardiac amyloidosis. Journal of the American College of Cardiology 2017;**70**(4):466-477.

25. Syed IS, Glockner JF, Feng D, Araoz PA, Martinez MW, Edwards WD, Gertz MA, Dispenzieri A, Oh JK, Bellavia D. Role of cardiac magnetic resonance imaging in the detection of cardiac amyloidosis. JACC: Cardiovascular Imaging 2010;**3**(2):155-164.

26. Ternacle J, Bodez D, Guellich A, Audureau E, Rappeneau S, Lim P, Radu C, Guendouz S, Couetil J-P, Benhaiem N. Causes and consequences of longitudinal LV dysfunction assessed by 2D strain echocardiography in cardiac amyloidosis. JACC: Cardiovascular Imaging 2016;**9**(2):126-138.

27. Damy T, Costes B, Hagège AA, Donal E, Eicher J-C, Slama M, Guellich A, Rappeneau S, Gueffet J-P, Logeart D. Prevalence and clinical phenotype of hereditary transthyretin amyloid cardiomyopathy in patients with increased left ventricular wall thickness. European heart journal 2015;**37**(23):1826-1834.
